# Barriers to Equitable Public Participation in Health-System Priority Setting Within the Context of Decentralization: The Case of Vulnerable Women in a Ugandan District

**DOI:** 10.34172/ijhpm.2020.256

**Published:** 2020-12-26

**Authors:** S. Donya Razavi, Lydia Kapiriri, Julia Abelson, Michael Wilson

**Affiliations:** ^1^Centre for Health Economics and Policy Analysis, McMaster University, Hamilton, ON, Canada.; ^2^Department of Health, Aging and Society, Centre for Health Economics and Policy Analysis, McMaster University, Hamilton, ON, Canada.; ^3^Department of Health Research Methods, Evidence, and Impact (HEI), Centre for Health Economics and Policy Analysis, McMaster University, Hamilton, ON, Canada.; ^4^Department of Health Research Methods, Evidence, and Impact (HEI), McMaster Health Forum, Centre for Health Economics and Policy Analysis, McMaster University, Hamilton, ON, Canada.

**Keywords:** Health System, Priority Setting, Public Participation, Vulnerable Populations, Decentralization, Uganda

## Abstract

**Background:** Decentralization of healthcare decision-making in Uganda led to the promotion of public participation. To facilitate this, participatory structures have been developed at sub-national levels. However, the degree to which the participation structures have contributed to improving the participation of vulnerable populations, specifically vulnerable women, remains unclear. We aim to understand whether and how vulnerable women participate in health-system priority setting; identify any barriers to vulnerable women’s participation; and to establish how the barriers to vulnerable women’s participation can be addressed.

**Methods:** We used a qualitative description study design involving interviews with district decision-makers (n=12), sub-county leaders (n=10), and vulnerable women (n=35) living in Tororo District, Uganda. Data was collected between May and June 2017. The analysis was conducting using an editing analysis style.

**Results:** The vulnerable women expressed interest in participating in priority setting, believing they would make valuable contributions. However, both decision-makers and vulnerable women reported that vulnerable women did not consistently participate in decision-making, despite participatory structures that were instituted through decentralization. There are financial (transportation and lack of incentives), biomedical (illness/disability and menstruation), knowledge-based (lack of knowledge and/or information about participation), motivational (perceived disinterest, lack of feedback, and competing needs), socio-cultural (lack of decision-making power), and structural (hunger and poverty) barriers which hamper vulnerable women’s participation.

**Conclusion:** The identified barriers hinder vulnerable women’s participation in health-system priority setting. Some of the barriers could be addressed through the existing decentralization participatory structures. Respondents made both short-term, feasible recommendations and more systemic, ideational recommendations to improve vulnerable women’s participation. Integrating the vulnerable women’s creative and feasible ideas to enhance their participation in health-system decision-making should be prioritized.

## Background

**Key Messages**

** Implications for policy makers**

- Participation in priority-setting processes can enhance the fairness and legitimacy of priority setting decisions by supporting the consideration and inclusion of a breadth of values, perspectives, and needs, including those of vulnerable women.
- Several barriers hinder vulnerable women’s participation in health-system priority setting within districts in Uganda. There is a need for policy- and decision-makers to change their approach to participation by understanding why women do not participate and recognize the root causes of these barriers.
- Vulnerable women can be meaningfully engaged to participate and also propose feasible mechanism to facilitate their participation.

** Implications for the public**

Public participation in health-system priority setting has been thought to contribute to increase accountability, enhance acceptability of the decisions, ease the processes of implementation, and ensure that the needs of different groups are considered when health system priorities are set. The public can provide local perspectives which can strengthen decision-making and enhance the acceptability of the decisions. Existing participatory structures in Uganda are meant to enable the public to participate in governmental decision-making. However, our results confirm earlier findings that vulnerable groups, specifically rural women, do not actively participate. The integration of women’s ideas to address barriers and enhance their participation in health-system decision-making should be prioritized concurrently with the implementation of top-down strategies.

Decentralization was used as an instrument to reconfigure the formal institutional structures in many countries around the world during the 1980s and 1990s.^1,2^ Decentralization takes different forms: devolution, deconcentration, delegation, and privatization.^3,4^ Each of these forms of decentralization has had implications for health reforms regarding the nature of the accountability relationships between local decision-makers (or constituents) and central government.^2,3^

 In Uganda, the 1995 Constitution and the Local Government Act 1997 devolved decision-making through the development of political structures called local councils (LCs) at the district, county, sub-county, parishes, and village levels^3,5,6^ (Figure). The LCs are responsible for their own elections, raising their funds, and have the authority to make budgeting decisions.^1^ Since the LCs are composed of elected representatives, they should be accountable to their electorate.^1,2,6,7^ The constitutional mandate for participation is quite broad referring to active participation of all citizens in governmental decision-making either individually or through representatives and civic organizations, without further elaboration about how this participation should be operationalized.^5-8^ While decentralization has improved public participation in political decision-making,^1,9-11^ the degree to which it has contributed to improving the participation of vulnerable populations in heath-system priority setting has not been well-explored. Our paper seeks to address this gap.

**Figure:**
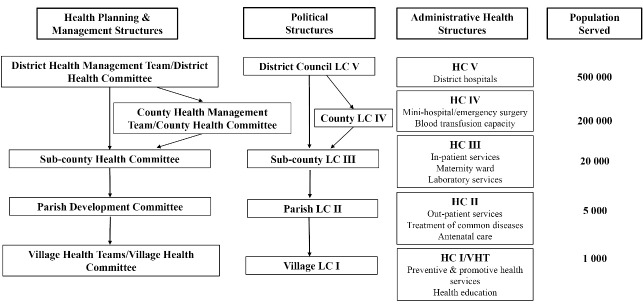
District Political Structures and Parallel Health Structures Under Decentralization.^6,11^ Abbreviations: LC, local council; HC, health center; VHT, Village health team.

 Like the political system, decentralization in the health sector involved the devolution of the responsibilities for planning, budgeting, and implementation of the health policies from the national level to District Local Governments.^6,7,12^ Decentralization of responsibilities for healthcare resulted in the promotion of public participation to enhance transparency and inclusiveness.^5,13^ According to the Ministry of Health documents, the public can participate in priority setting either directly (eg, in community-based activities such as public health campaigns or by attending village council meetings),^10,12^ or indirectly through publicly elected representatives.^10,12^ Health-system decision-making occurs at the sub-county and district levels. Each LC level has a corresponding health committee.^5,11,14^ The health committees are responsible for health-system planning, budgeting, monitoring and evaluation.^12^ They also contribute to fostering community participation in decision-making.^5^ Health unit management committees were developed, in part, to facilitate community participation in decision-making within the health units.^10,15^ At the village level, village health teams (VHTs) were developed to improve community ownership and responsibility for the implementation of health promotion and prevention campaigns and community health education. VHTs also function as a link between the community to their primary health unit, encouraging communities to participate in the management of their local health services.^11,12^ However, guidance around explicit standards for effective participation and operationalization of participation remain unclear.

 One function of the devolved political structures is to set health system priorities. Priority setting is a process through which decisions about resource allocation between competing programs are made.^16^ Participation in priority setting contributes to ensuring that the unique needs of different groups are considered. Participation can also contribute to equitable health-systems.^17,18^ While the public may lack technical health knowledge, they can provide information, which is relevant to the priority-setting processes.^17-19^ Considering public values contribute to improving the quality of the decisions, their acceptability and feasibility.^17,20-23^ Furthermore, it is thought to promote decision-maker accountability.^19,24,25^ One subset of the public that appears not to be participating in health-system priority setting are vulnerable groups.^26^

 Vulnerability is a complex construct that is often used interchangeably with concepts such as “marginalized” or “disadvantaged.” Discussions of vulnerability in the wider bioethics’ literature highlight that lack of power, agency and autonomy makes some individuals more susceptible to exploitation.^27-29^ The Uganda Human Development Report (2015) echoes this by defining vulnerability in terms of (*i*) exposure (which relates to susceptibility to risk), and (*ii*) resilience (which relates to the available choices and ability to deal with the exposure).^30^ Addressing the health concerns and priorities of vulnerable groups is essential to health equity, since their needs may be different and more severe or urgent than those of the general public.^31,32^ Ugandan policy documents identify the vulnerable populations as: women, widows, orphans, children, adolescents, the elderly, people with disabilities, displaced persons, and people living in chronic poverty.^12,33,34^ They also recognize that vulnerability varies according to gender, age, ethnicity, occupation, and social status,^35^ and recommend affirmative action for traditionally marginalized groups, such as women.^7,34^ Individuals and populations can experience multiple, interacting layers of vulnerability that compound their risks.^36^ Vulnerability exists on a spectrum and is a dynamic condition rather than a permanent fixture of a person or populations, rather vulnerability can be context-dependent.^27,28^ For example, in Uganda, elderly women can be perceived as both vulnerable and empowered. Elderly Ugandan women are often likely to be widows and become economically dependent on others,^37^ at the same time, elderly men and women experience a relatively high status in Ugandan society and are well-respected in the community.^38^

 Exclusion of vulnerable women from health-system decision-making can mean that their perspectives and health needs are not integrated into health-system priorities. Women are a central part of the Ugandan health system both as caregivers and as patients themselves.^39^ As evidenced in the literature on healthcare in Uganda, multiple social stratifiers including ethnicity,^40^ disability,^41^ economic status and class,^39,42^ rurality,^43^ and age,^42^ intersect with gender to further exacerbate gender gaps in health service access and health status in the country. It has been argued that, especially in low- and middle-income countries, gender power relations create inequities both in access to resources and decision-making.^40^ When priority-setting processes do not consider the perspectives of vulnerable women, their interests may be further marginalized, leading to even greater health disparities.^31^ Vulnerablilty of Ugandan women arguably stems from both asymmetry of power in the household, workforce, and society at large and the intersection of the above mentioned social stratifiers. This hinders their participation in decision-making.^44^ To overcome this, Uganda’s National Gender Policy compels all government policies and programs to work towards elimination of gender inequalities.^45^ The policy provides guidance for gender mainstreaming and women’s participation in governance, decision-making, planning, resource allocation, and implementation of development programs.^45^ The Local Governments Act 1997 mandates one-third representation of women across all levels of governance.^6^ Therefore, we would expect that women are being sought out to participate in health-related decision-making, including priority setting. In practice, however, community participation and empowerment has been limited thus far.^11,14^

 While the focus of this study is on vulnerable women’s participation, most of the literature on public participation does not segregate the vulnerable groups.^13,17,18,23,24,46-48^ The meagre literature that specifically talks about vulnerable populations has highlighted the relevance of their participation^49,50^ and the need for strategies to engage disadvantaged, marginalized, or vulnerable groups, in priority setting.^24^ Some of this literature identifies lack of participation for vulnerable populations and offer potential explanations. For example, McCollum et al explored power dynamics and participation in health-system priority setting following devolution in Kenya and found that power imbalances resulting from underlying social structures perpetuate the exclusion of the most vulnerable from priority-setting processes.^51^ However, these studies focused on the perspectives of decision-makers rather than those of vulnerable populations. Studies of the feasibility of the methods proposed to operationalize public participation in high-income countries,^46^ and low-income countries such as in Tanzania,^22^ demonstrate that public participation helps shape the priorities that are set, however explicit participatory methods are required to support meaningful public participation. These studies re-emphasize the difficulties with operationalizing systematic public participation, highlighting the challenges related to involving vulnerable populations.^22,46^ However, these studies did not identify the factors that hamper the participation of the vulnerable populations. Furthermore, the literature about vulnerable populations’ participation in health-system priority setting in Uganda is also limited. Even in countries where governments’ ascribe to public participation, like Uganda, Kapiriri et al found that vulnerable populations’ participation is lacking^5^. While this study identifies barriers that may hamper these groups’ participation, an in-depth examination of vulnerable women’s participation is missing.^5^

 Our study seeks to address these gaps by examining the participation of vulnerable Ugandan women in health-system priority setting. We explored the role of vulnerable women in health-system priority setting within the context of decentralization in a rural district in Uganda. Specifically, we aimed to: (*i*) examine self-reported and decision-maker reported vulnerable women’s participation in health-system priority setting within the district; (*ii*) identify the barriers to vulnerable women’s participation; and (*iii*) establish how the barriers to vulnerable women’s participation might be addressed.

## Methods

###  Study Design

 We used a qualitative description design.^52,53^ Qualitative description provided the opportunity to explore the phenomenon of participation of vulnerable women in health-system priority setting, within the specific context of a rural district in Uganda, using different data sources.

###  Study Setting

 We selected Uganda primarily because is it a low-income country operating within the context of decentralization, which has implemented participatory structures at each level of government.^7^ The most recent census from 2014 reports that the Uganda has a population of 34.9 million people with a sex ratio of 94.5 males per 100 females. The country is divided into 111 districts and one city, Kampala. It has been estimated that 72% of the population lives in rural areas.^33^ This study was conducted in a typical rural setting in Uganda, the Tororo District, where we may expect to find women who are especially vulnerable. Since the district has one of the highest poverty levels in the country,^54^ we expected to access the vulnerable population of interest with ease. Tororo district is made up of 17 sub-counties and 2 major ethnic groups, the Jopadhola and the Iteso. Jopadhola are the population majority in the district. While the 2 ethnic groups have lived alongside one another peacefully for decades, recently tensions have emerged as the minority Iteso advocate for the creation of their own district.

###  Study Population

 We interviewed both vulnerable women living in rural communities and decision-makers. Vulnerable women were sampled to reflect the relevant dimensions of vulnerability such as age, ethnicity, education, marital status, and employment. Decision-makers included the sub-county leaders and members of the district health management team (DHMT), who are responsible for health planning, organizing, monitoring and evaluation of services within the district.^33^

###  Sampling 

 Four sub-counties were sampled including: one Jopadhola dominant, one Iteso dominant, and 2 mixed ethnicity sub-counties. The sub-counties were geographically dispersed to represent the western, central, and eastern regions of the district.

 Initial sampling of the vulnerable women involved snowball sampling,^55^ whereby in each sub-county an index woman was identified in any of the age groups of interest: adolescent/young adult (10-24 years), adult (25-55 years), and elderly (55+ years). After their interview, the index respondent was asked to refer us to any additional women within their sub-county, who met the age criterion. Once we achieved saturation across the following dimensions of interest: desire to participate, whether and how they participate, barriers to participation, and recommendations to enhance participation, we tallied the vulnerabilities that were represented (ethnicity, level of educational attainment, marital status, and income/type of employment) to identify vulnerabilities that may be lacking in the sample. Purposeful sampling was used to identify additional respondents to ensure saturation in all dimensions of vulnerability.

 For the decision-makers, we interviewed all members of the DHMT (district level) and purposeful sampling was used whereby we interviewed technical leaders (sub-county level) who had specialized health knowledge and/or were involved in sub-county planning, budgeting, and decision-making processes.

###  Data Collection

 In-depth interviews were conducted using pilot-tested, semi-structured interview guide developed based on themes from the literature on participation in priority setting and Ugandan policy documents. SDR conducted all the interviews. While decision-maker interviews were conducted in English language, interviews with the vulnerable women employed a translator who translated the English questions to the local languages and back translated the women’s responses to English for SDR. All interviews were audio-recorded, with permission from the participants. Examples of questions from the interview guide for vulnerable women included: “How are decisions about healthcare made in your community?”; “In what ways are you involved in decision-making processes about the health system in the district? At the village level?”; “How do you believe the district can improve the participation of women in decision-making about resource allocation in the health system?” (Supplementary file 1). Whereas, sample questions from the interview guide for district decision-makers included: “Tell me about participation within the district?”; “Who are considered vulnerable women in Tororo District from the perspective of the district?”; “How do these vulnerable women participate in priority setting decisions at the local level?”; “How should these women be involved in making decisions about the distribution of resources?” (Supplementary file 2).

###  Data Analysis

 All interviews were transcribed and back translated by Ugandan transcriptionists with expertise in the local languages and English. Interviews with the DHMT and sub-county leaders were transcribed verbatim. QSR NVivo12 qualitative data analysis software was used to code interview transcripts. We used an editing analysis style, which supports an inductive approach to data analysis and grounding of emerging concepts in the data.^56,57^ In the initial phase, we conducted line-by-line reading of the interview transcripts and used an open, inductive stance to microcode 5 interviews from each respondent group (vulnerable women and district decision-makers). Similar ideas were grouped together and given a concept label.^58^ In the second phase, we used a deductive approach and applied the generated concept labels to code the rest of the interviews,^58-60^ while also pursing emerging concepts for theoretical variation to the point of saturation.^61^ Specifically, we continued to seek out participants and conducting interviews until no additional insights on vulnerable womens’ participation appeared in the data, subsequently we conducted an additional 4 interviews to ensure stable saturation. Comparison to the literature confirmed that relevant conceptual categories had been identified and explored thoroughly.

## Results

 We interviewed total of 57 respondents including 12 district level (3 women, 10 men), and 10 sub-county decision-makers (2 women, 8 men); and 35 vulnerable women. All 12 members of the DHMT were interviewed, while sub-county level respondents included sub-county chiefs, community development officers, secretaries for health, and health inspectors. The vulnerable women’s characteristics are summarised in Table 1.

**Table 1:** Demographic Information for the Vulnerable Women Interviewed (N = 35)

**Demographic Characteristics/Vulnerabilities of interest**	**Adolescent/Young Adult** **10-24 Years ** **(n = 11)**	**Adult** **25-54 Years ** **(n = 12)**	**Elderly** **55+ Years ** **(n = 12)**	**Total**
Ethnicity				
Jopadhola	3	5	4	12
Iteso	6	5	6	17
Jopadhola-speaking Iteso^a^	2	2	1	5
Other	0	0	1	1
Education				
None	0	1	5	6
Primary	8	7	6	21
Secondary	3	3	1	7
Post-secondary	0	1	0	1
Marital status				
Single (ie, never married)	6	0	0	6
Married	3	9	4	16
Divorced	2	1	0	3
Widowed	0	2	8	10
Employment				
Unpaid work (including subsistence farming)	9	5	8	22
Paid work (including petty business eg, farming for sale, handcrafts, hair stylist)	2	7	4	13

^a^Jopahdola-speaking Iteso lived in a Jopahdola dominant sub-county.

 The following section is organized according 4 dimensions of participation examined in this study: desire to participate, actual participation, barriers to their participation, and recommendations to enhance their participation health-system priority setting. We report on the different perspectives of vulnerable women’s participation using the following labels: “ethnic group, age” for the vulnerable women, “sub-county leader” for the sub-county level decision-makers, and “DHMT member” for the district level decision-makers.

###  Perceptions of Participation for Vulnerable Populations 

 There was consistency in responses from the vulnerable women and the decision-makers. Both groups of respondents stated hat vulnerable populations, including children, youths, the elderly, people with disabilities, and the very poor, do not participate. Both groups explicitly stated that vulnerable women, do not participate, and this vulnerable group is the focus of this paper.

 However, perceptions of vulnerable women’s participation varied depending on the administrative level where participation was to occur rather than the category of respondent (vulnerable women or decision-makers). At the community level, both district decision-makers and vulnerable women agreed that women and powerful men (ie, local leaders), participated. The vulnerable women reported more active participation than men in both formal and informal meetings, including LC and community meetings. According to the vulnerable women, however, more women than men attend informal community meetings.

 Beyond the village, vulnerable women and district decision-makers agreed that men participate more and are more represented at all levels of the decision-making structures than women. District and sub-county level decision-makers highlighted identified that vulnerable women were either underrepresented or not engaged in health-system priority setting within the district. They explained that while women should be represented, at all levels, they are generally missing at planning meetings like the annual budget conferences. We have included a sample agenda for a district level budget conference, where all members of the public, including vulnerable women, are meant to participate in the open discussion that occurs following each committees’ presentation (Supplementary file 3). When asked about vulnerable women’s participation at the district level, one member of the DHMT specifically identified that women do not participate in district budget conferences. “… Most times the woman in the village does not get that opportunity.”

###  Do Vulnerable Women Want to Participate?

 The vulnerable women interviewed expressed a desire to be engaged. They believed they could make valuable contributions since they understood their communities and had knowledge of community needs. For example, one respondent pointed to the role of elder women in their village as experts about the needs of their community:

*“You can get more ideas from those elders, they will advise you how to go with people, if you have not understood how to organize the community. Those elders know how, they have lived there for long…” *(Iteso, 50).

###  Barriers to Participation for Vulnerable Women

 Respondents identified twelve key barriers to the women’s participation. These were grouped into 6 overarching categories: financial (transportation and lack of incentives to participate), biomedical (illness/disability and experiencing menstruation), knowledge-based (lack of knowledge, namely general education, literacy, and English language skills, and lack of information about rights and opportunities to participate), motivational (perceived disinterest, lack of feedback, competing needs and time commitments), socio-cultural (lack of decision-making power for women), and structural (hunger and poverty).

 As illustrated in Table 2, all barriers were identified by both decision-makers within the district and vulnerable women, except for menstruation (identified by the adolescent girls only). Lack of information about their right to participate and opportunities to participate in health-system planning and budgeting meetings was a prominent barrier that was identified by the vulnerable women. As clarified by one 24-year-old Iteso woman, “*I am interested in meetings but what can prevent me from going is … if I have not got the information, I don’t go*.” For some vulnerable women, while they may hear about these meetings, lack of education deterred them from attending meetings. These women were concerned about their lack of English language skills or the usefulness of their contributions compared to those with higher education (Table 2). One 49-year old Japadhola woman remarked, *“There are questions they ask in English and if you are not educated, you cannot communicate.”* Another concern reported by both decision-makers and the vulnerable women was the lack of feedback and implementation of the promises that are made. Furthermore, the women perceived that resources either remain at the district or are distributed elsewhere, but not in their villages. One 30-year old Iteso woman explained, “*They usually bring things at the district, but sometimes those things don’t reach here in the village….*” Additional illustrative quotes for each of the barriers are presented in Table 2.

**Table 2:** Barriers to Participation for Vulnerable Women in Health Sector Priority Setting

**Category of Barrier**	**Barriers to Participation**	**Identified by**	**Illustrative Example(s)**
Financial	Transport (distance/cost)	• DHMT • Rural women	*“I don’t reach to the higher-level meetings… I don’t always attend because it is difficult for me to reach there, it is far”* (Iteso, 59).
	Lack of incentives/compensation for time	• Sub-county leaders • Rural women	*“They should be given something for motivation and if others see this, they will be encouraged to attend the meetings”* (Japadhola, 80).
Biomedical and/or health	Illness/Disability	• All	“I* was willing to continue [participating] but I have a problem with my leg I cannot walk easily”* (Japadhola, 80).
	Menstruation	• Rural women	“*Sometimes when I am in my menstruation period, I stay at home … (due to) absence of pads [sanitary napkins]… I miss, I don’t go”* (Japadhola, 16).
Knowledge-based	Lack of education (knowledge/literacy)	• All	“S*o even if you go there you find people who come who ended in P.7[seven years of schooling], S.4 [eleven years of schooling], S.2 [nine years of schooling] and for you who have never gone to any level you can understand anything*” (Iteso, 57). *“If the person cannot read and write or speak English because it (the meeting) is conducted in English, most times the woman in the village, does not get that opportunity*” (DHMT member).
	Lack of information about participation (rights/opportunities)	• All	“*Because they are not informed, they cannot know that a meeting is going to take place, but if they were informed, they would go”* (Japadhola, 52).
Motivational	Perceived laziness/disinterest	• Sub-county leaders • Rural women	“*People in the village are lazy*” (Iteso, 16). “*People who are drunk are married to alcohol, they don’t want to listen to the chairman or to the people who come*” (Iteso, 59).
	Competing needs/time commitments	• All	“*Women from the village cannot go to the sub-county, because there are competing time needs like; digging, cooking … for me who is a widow, I have a lot of responsibility”* (Iteso, 57).
	Lack of feedback/follow through	• Sub-county leaders • Rural women	*“Things are supposed to be here (in the village) but they don’t reach to people at their homes, sometimes you hear that they have given out things like medicines, but here people don’t get them … they should not just come once and never come back, they should always be coming*” (Iteso, 30). “*Personally, my idea is that whatever has been discussed should not remain here, it should be put in practice*” (Japadhola, 18).
Socio-cultural	Lack of decision-making power	• All	“*You may find women may be too interested in being involved but they can’t come or their husbands do not allow them to come in for the meetings so it’s more of the power relation bit in a home where the man says I am going to attend the meeting, you don’t need to go”* (Sub-county leader). “*Culturally the women are not supposed to attend the meetings. Culturally, the woman is not supposed to be heard” *(DHMT member).
Structural	Hunger	• All	“*When somebody comes trying to ask them questions and if someone slept hungry, they will not be able to talk to you”* (Iteso, 50).
	Poverty	• All	“*The very poor, when you tell them to come, they [may] attend meetings but after the meeting … they want something [money]”* (Iteso, 60).

Abbreviation: DHMT, district health management team.

 There was a coincidence of views between the 2 types of respondent since district decision-makers reiterated many of the barriers identified by the vulnerable women. For example, when discussing participation at budget conferences barriers such as lack of education or English literacy and lack of information about opportunities to participate were identified.

*“The budget conference is attended by various stakeholders of the district, religious leaders, cultural leaders, other implementing partners, NGOs [non-governmental organization], CBOs [community-based organizations], faith-based organizations, we have the civil society, the press, the business community, everybody. Who is not there... Is the person who cannot read and write or speak English because it is conducted in English” *(DHMT member).

*“It’s an open thing but the problem is are people aware about the budget conference? First off, information does not reach many, you find that some of them think that even if they come, their views may not be listened to, who am I, that’s the question, even me the poor woman in the village if I go there to the district level, who will recognize me. That’s another big problem, they have somehow given up, who will listen to us... I think it’s some kind of inferiority complex. Then thirdly I think this meeting is held at district level headquarters, how do you expect somebody to travel from [one of the furthest sub-counties from the district headquarters]”* (DHMT member).

 Furthermore, often there is not simply a single barrier that prevents women from participating, but multiple interacting barriers. For example, one adolescent mother explains:

*“When my baby is sick like last time when I was supposed to go to a meeting, I had no transport, so it was difficult for me to go from here to the meeting place on foot with the baby” *(Iteso, 16).

 We found that many of the identified barriers are inter-related. For example, while the vulnerable women respondents strongly emphasized lack of transportation, hunger, and lack of incentives to attend meetings as direct barriers to participation, these are also symptoms of poverty. While this demonstrates the complexity of including vulnerable women in participatory processes for health-system priority setting, planning, and budgeting, it also provides for the opportunity to develop holistic solutions that address multiple barriers.

###  Recommendations From the Field

 Two types of recommendations were made by our respondents: (1) specific strategies to tackle the barriers to participation (Table 3), and (2) general strategies to enhance participation for vulnerable women across the district.

**Table 3:** Specific Strategies to Address Barriers to Participation for Vulnerable Women in Health System Prioritization Processes

**Category of Barrier**	**Barriers to Participation**	**Recommendations From the Field**
Financial	Transport (distance/cost)	Hold meetings at the health centers within the community*
	Lack of incentives	Provide incentives including transport, allowance, food*
Biomedical and/or health	Illness/Disability	Provide transportation*
	Menstruation	Provide adolescent women with female hygiene products*
Knowledge-based	Lack of knowledge (education/literacy)	Hold meetings in local language and/or provide interpreter services*
	Lack of information about participation (rights/opportunities)	Identify an enthusiastic, capable woman from the community sensitize and educated about participation. She would return to collaborate with the community
Motivational	Competing needs/time commitments	Host meetings at times when target populations can attend*
	Perceived laziness/disinterest	The women already gather informally, add a formal representative to meetings*
	Lack of feedback/follow through	Strengthen community dialogues/barazas to enhance two-way communication between rural women and local governments
Socio-cultural	Lack of decision-making power	Develop and support females within the local governance structures
Structural	Hunger	Organizers provide some type of snack or lunch*
	Poverty	Social assistance and development programs to target poverty and daily living expenses ie, school fees, adequate housing, skills training

* The asterisks identify immediately feasible strategies. Recommendations that are not followed by an asterisk are longer-term strategies.

 The vulnerable women made feasible recommendations to address hunger, transport, and lack of incentives, primarily involved compensation for the time spent and resources required to travel to and participate in planning and budgeting meetings. The need to support and feed their families (through farming) creates competing needs and time commitments that hinder their ability to participate. These challenges are exacerbated by lack of transportation and the cost associated with travelling long distances. Practical recommendation from the field to overcome these barriers was to ensure that meeting times fit women’s schedule and that they were held within the communities, for example at local health centers, rather than at the sub-county headquarters.

 Our respondents reported 2 general strategies for facilitating public participation over the long-term, (*i*) improving channels of communication, and (*ii*) developing and implementing economic and social empowerment initiatives.

 Firstly, the vulnerable women expressed that this lack of follow-up with communities by decision-makers about resources allocation decisions may result from poor communication between the village and the district. While the LC system was established to, in part, facilitate communication, our respondents believe that these structures are not functioning as they should.

*“The district is too large for me to say maybe they come down to meet the local women, it might be very, very difficult, that is why they are using the other system of LC … [but] there is communication barrier”* (Iteso, 55).

*“Any decisions made… through the sub-county, parish and then village, they [should] follow the channels of communication [but] they don’t follow”* (Japadhola, 50).

 Secondly, improved economic and/or social empowerment was a recommendation made by the district decision-makers and the rural women. Poverty was perceived as the root of many other barriers. They explained that when the country is experiencing periods of drought and famine, populations are hungry and experience illnesses, citizens lack adequate shelter and housing, parents cannot afford children’s school fees, people do not have money for transportation, and women lack decision-making power in the home. This makes participation in health-system decision-making a lower priority.

*“Like this time of famine, you find that someone did not eat and has no energy … Another can be sick and is admitted (to hospital) and cannot attend the meeting…” *(Japadhola, 49).

*“We were many [children], so my father decided to educate the other ones and say a girl child, you need not to go school, your work is to get married” *(Iteso, 38).

 When asked to consider the barriers they identified and strategies to improve participation for vulnerable women, the vulnerable women respondents suggested that women need to become empowered through social assistance and skills development programs (Table 3). For example, one vulnerable woman explained:

*“So I think if they can only get them kind of a cooperative, we used to have cooperatives of farming, or cotton… and the money is given to her and she simplifies also her life, at least gets soap, salt, those simple things for their basic requirements”* (Iteso, 50).

 These sentiments were echoed by the decision-makers, as illustrated by the following:

*“Now we have got the youth livelihood program where government is giving them money … for example a group of youth of about ten or fifteen youth in one community will say we are interested in rearing cows for milk, then they apply at the district level, then they are given money … it’s supposed to improve livelihood and also reduce on this issue of non-employment” *(DHMT member).

 The respondents felt these strategies would strengthen their capacity and motivation to participate in health-system planning. Additional illustrative quotes for each of the recommendations can be found in Supplementary file 4.

## Discussion

 Both vulnerable women and district decision-makers reported that women living in rural Uganda were not consistently participating in health-system priority setting at all levels within the district. Powerful stakeholders like political leaders and technical experts participate in district priority setting. While vulnerable women expressed a desire to participate, there are numerous barriers that prevent their participation (Table 2). Our respondents provided ideas about how to best address these barriers to enhance participation for women within the district (Table 3).

 The findings that vulnerable groups, and women in particular, do not participate in decision-making at all administrative levels are consistent with the few other studies in the field^5,17,24,62^ and specifically in health sector priority setting in low- and middle-income countries.^13^ While the vulnerable women seem to be participating more at the community/village level, decision-making occurs at the sub-county and district levels, where they do not participate due to the various barriers.

 The barriers consistently identified by both types of respondents are not unique to Tororo district or Uganda. For example, lack of decision-making power, specifically for women were reported in Mukono district, Uganda,^5^ while lack of information and time constraints have been reported in Kenya^63^ and Tanzania.^64^ The desire for financial compensation and other incentives to participate such as providing food or transportation fees have been reported elsewhere and are symptoms of poverty, a prominent barrier identified both respondent groups in this study.^5,13,48^ Menstruation was the only barrier that was not consistently reported by both types of respondents and only identified by adolescent girls. Menstruation is a more immediate and relevant barrier for adolescent girls when compared with adult women especially since they are economically dependent on the adults in their household to obtain menstrual hygiene products.^65^

 The Ugandan government has stated its commitment to women’s participation.^6,7,45^ Yet, post decentralization, the structures meant to enhance participation in health-system planning and priority setting do not seem to be functioning as intended.^5,11^ For example, these structures were not identified by the vulnerable women as avenues they used to participate. This is consistent with literature that explains that the existing structures have not facilitate the participation of vulnerable populations.^5,10^ Nevertheless, we found that women gather informally at the village level to voice their concerns, which negates the notion that women are lazy and disinterested. The other barriers discussed above may be contributing more to the women’s lack of participation in formal decision-making.

 For priority setting to be truly bottom-up, all publics should be able to access the participatory structures. When engaging vulnerable populations in health-system decision-making, transparency and accountability are necessary to maintain community interest.^5,13,66^ The onus is often place on decision-makers when priorities are not set with community input or according to community needs. However, the decision-space for district leaders to set health-system priorities may be limited,^5,67^ restricting their ability to be responsive to community needs. Furthermore, lack of participation for vulnerable women may not be a function of a failure in decentralization, rather it may be practically difficult for local leadership to access these women, considering the district’s limited resources.^68^ Our study offers insight into opportunities for decision-makers to use existing decentralized structures to encourage vulnerable groups’ participation in health-system priority setting. While systemic and structural recommendations to support poverty alleviation are needed, these strategies may be more ideational in nature. Our respondents’ recommendations support the development of more immediate and feasible strategies to improve vulnerable women’s participation. For example, consistent dialogue with communities provides opportunities to explain and justify the decisions in ways that are acceptable, and this is especially important when resource allocation does not align with public input. Feedback about programming is essential to maintain a trusting relationship with communities.^5,13,48^ One strategy identified in our study, community dialogues or barazas, are community-based fora for monitoring performance of programmes, including health programming, and acts as one platform for citizens to participate in the planning and development cycle. In these fora all citizens, including vulnerable women, can participate in monitoring the use of public resources as Local Governments delivery of services at the local level.^69^ Not only are barazas meant to facilitate monitoring and evaluation of project implementation, but they are also a mechanism for identifying priority areas for action.^69^ In the context of health system priority setting, barazas could provide a platform for a two-way dialogue involving well-informed and skilled representatives from the district, sub-county, parish, and villages levels of governance and the public, in addition to budget conferences and village meetings. Therefore, while the decision-space for district decision-makers may be limited, transparency and accountability between local leadership and communities may contribute to building trust, especially when the community’s felt needs cannot be addressed.

## Study Limitations

 There are some limitations to the study findings.

 The use of a translator and local language transcription and translation may have hampered the understanding and affected the meaning of the questions. Translator training, regular meetings to review the questions and responses as well as verbatim transcribing and back transcribing the interviews contributed to our belief that we obtained the respondents’ own ideas.

 Finally, we did not interview the health unit management committee, which is also meant to facilitate public participation. Since the focus of the study was on health systems, health units/ services were beyond the scope of the study.

## Conclusion

 Our study adds to the limited literature on engagement of vulnerable populations in health-system priority setting. Existing participatory structures in Tororo district are meant to enable public participation in governmental decision-making, from the village up to the district level. However, we found that vulnerable women are not actively participating at all levels. Women’s participation is localized to village meetings, most often informal meetings and discussions among friends and neighbors rather than the LC meetings. They rarely participate directly at higher administrative levels. This is problematic because policy-making and resource allocation decisions are made at the sub-county or district. While there are opportunities for participation at the sub-county, such as at budget conferences, direct participation for vulnerable women at the district may be impractical. Since participation at the district is meant to occur through representation facilitated by decentralization and mandated by the Ugandan Constitution and the Local Governments Act, future studies could explore whether representation is an effective mechanism for participation.

 The variety of practical and innovative recommendations to facilitate women’s participation discussed could be explored. Particularly, the recommendation to educate motivated women from the village about participation and train them as local leaders to promote participation in their communities is relatively feasible and could be implemented. This could create an environment of empowerment within the community whereby women can act as peer leaders and educators in the community, fostering relationships with women and other vulnerable populations and encouraging their participation.

 Decision-makers can change their approach to community engagement by understanding why women do not participate and recognize the root causes of these barriers. A two-way communication channel between communities and decision-makers is essential to women’s participation. Strengthening community dialogues would allow for mutual learning between these parties. The integration of women’s ideas, that are consistent with district priorities, would enhance their participation and should hence be prioritized concurrently with the implementation of top-down strategies.

## Acknowledgements

 We would like to thank our respondents from the Tororo district, Dr. David Okumu for facilitating access to the communities, and Harriet Akisa for assisting with translation during data collection.

## Ethical issues

 Ethics approval was obtained from the Hamilton Integrated Research Ethics Board (HiREB), Canada, and the Makerere University School of Public Health (MakSPH) Institutional Review Board, Uganda. All respondents were assured of the confidentiality of their responses and participation and informed of their right to withdraw from the study. They provided either written or thumbprint consent prior to participating in the interview. Key informants were not compensated, financially or otherwise, for their participation.

## Competing interests

 Authors declare that they have no competing interests.

## Authors’ contributions

 SDR was responsible for conceptualizing the study, with input from LK, and its methodological design, with input from all authors: LK, JA, and MW. SDR was responsible for data collection, analysis and drafting the manuscript. All co-authors provided feedback on several drafts, which were incorporated into the manuscript.

## Funding

 This work was supported by the Canadian Institute for Health Research, which supplied the funding for the corresponding author’s research scholarship.

## Supplementary files


Supplementary file 1. Semi-structured Interview Guide (Rural Women).

Supplementary file 2. Semi-structured Interview Guide (District Level Decision-Makers).

Supplementary file 3. Programme For Tororo District Local Government.

Supplementary file 4. Recommendations to Address Barriers and Enhance Participation for Vulnerable Women in Healthcare Prioritization and Planning Processes.
