# Supplementary material for: Barriers to Equitable Public Participation in Health-System Priority Setting Within the Context of Decentralization: The Case of Vulnerable Women in a Ugandan District
Source: Int J Health Policy Manag. 2020 Dec 26;11(7):1047–57. doi: 10.34172/ijhpm.2020.256 (PMC9808191; doi:10.34172/ijhpm.2020.256)
Supplement: Supplementary file 1 — Semi-structured Interview Guide (Rural Women). [file ijhpm-11-1047-s001.pdf]

Supplementary file 1. Semi-structured Interview Guide (Rural Women)

Participation of Vulnerable Women in Priority Setting Processes in a Rural District of Uganda

Background

I would like to thank you for agreeing to participate in this interview. I would also like to remind you that if there are any questions that make you uncomfortable and you would prefer not to answer, you are free to decline answering. You are also free to stop the interview and withdraw from the study at anytime. If you choose to do so, all you have to do is let me know that you no longer wish to continue. If you have any questions at any point throughout the interview please do not hesitate to ask for clarification.

Let us start with an example. If food is limited and all members of your family fall ill, who would you choose to give the food to first, and who would you decide to get medical attention for first? In health care, decisions need to be made in a similar way. Options are ordered and people need to make decisions about what is most important, and what cannot be done due to lack of resources. The purpose of this study is to explore and understand involvement of women like you in making decision about how resources should be distributed in your health system. I'd like to learn about your perspectives in order to understand how you can meaningfully participate and provide input into these types of decisions.

Are you comfortable to proceed?

Background Demographics

In order to provide some context, I will ask you to tell me a bit about yourself.
[Probes]

- How old are you?
- Which ethnic group do you identify with?
- Are you married? Do you have children? If so, how many?
- Tell me about your education?
- Tell me about your work/occupation? [Your spouse's work?]

General: Community level decision making

- Do you know how decisions about healthcare are made in your community? Tell me about this process.
- Who is involved in these processes? How are they involved?

- Who is not is not involved? Can you tell me about the reasons that you believe they are not involved?

  - [Probes] vulnerabilities including age, gender, socioeconomic status
  - What are some of the barriers that you think prevent their involvement?

    - [Probe] financial, physical factors, etc.

General: Participation

- In what ways are you involved in decision making processes about the health system in the district? At the village level?
  [Probes]

  - Structures for participation
    - Can you tell me about participation at the Budget Conference?
    - I am aware that budget conference occur both at the district and the sub-county level, can you tell me a bit more about at what level you would participate and how?
    - Are there other structures that you participate in? Ie. Involvement with Village Health Teams (VHTs)

  - How are you made aware of these opportunities?
    - Ie. Radio announcements? Others?

- To what extent are you satisfied with your level of involvement in decision making about where resources are allocated in your health care system?
  - What are the barriers to your involvement? How would you rank these barriers based on their importance to you?
  - What can be done to improve your level of satisfaction with your involvement?

- What factors are important to you and do you think should be considered when making decisions about resource allocation within the health system?
  - Provide example(s)

Participation: What could your contribution be?

- What do you believe you can contribute to the decision making process about how resources for health are allocated within district?

- What do you feel would be the benefit of including your perspectives or perspectives of people similar to you in decision making?

Participation: How?

- How do you think that your perspectives about where resources should be allocated and your health needs can be better communicated to decision makers?

    - What if it is not possible personally attend meetings like budget conferences where resource allocation decisions are made, how can your interests and needs be represented?

- How do you believe the district can improve the participation of women in decision making about resource allocation in the health system?
    - [Probe] Based on your identification that …. Are not participating, how do you believe the district can improve participation for them?

- Is there anything further that you wish to add?

Thank you for your participation.
