# Supplementary material for: Barriers to Equitable Public Participation in Health-System Priority Setting Within the Context of Decentralization: The Case of Vulnerable Women in a Ugandan District
Source: Int J Health Policy Manag. 2020 Dec 26;11(7):1047–57. doi: 10.34172/ijhpm.2020.256 (PMC9808191; doi:10.34172/ijhpm.2020.256)
Supplement: Supplementary file 2 — Semi-structured Interview Guide (District Level Decision-Makers). [file ijhpm-11-1047-s002.pdf]

Supplementary file 2. Semi-structured Interview Guide (District Level Decision-Makers)

Participation of Vulnerable Women in Priority Setting Processes in a Rural District of Uganda

Background

I would like to thank you for your willingness to participate in this interview. If there are any questions that make you uncomfortable and you would prefer not to answer, you are free to decline answering. You are also free to stop the interview and withdraw from the study at anytime. If you choose to do so all you have to do is let me know that you no longer wish to continue. If you have any questions at any point throughout the interview please do not hesitate to ask for clarification.

The purpose of this study is to explore and understand participation of vulnerable women in priority setting processes in Uganda. Priority setting is a process through which choices are made about how to distribute resources. Any health care system requires priorities to be identified and from the list of possible priorities, decisions need to be made about how resources are distributed between competing health programs, services, and drugs delivered with health systems. The resource allocation process involves making choices about where resources are allocated based on ordering items in terms of importance with justifications for those choices. I am hoping to learn about your perspectives in order to understand how vulnerable women can meaningfully participate and provide input into decision making about resource allocation decisions.

Are you comfortable to proceed?

Background Demographics

In order to provide some context, will you tell me about yourself and your role in the DHMT? [Probes]

- What are some of your major responsibilities as [Participant's Title]?
- How long have you served as a member of the DHMT?
- Can you tell be about your education prior to working at the DHMT?

General: Decision Making

- How are resource allocation decisions made at the district level?
  [Probe]
    - Role: the executive, district council, standing committees
- From your perspective, how does the district define vulnerability?
    - Who is considered vulnerable from the perspective of the district? Why?

- How are decision makers informed about the health needs of vulnerable groups?

General: Participation

- Can you tell me about participation within the district?
    - [Probes]:
        - Who participates directly in decision making for resource allocation in health care?
        - What are some of the barriers to participation?
        - Who represents those who do not participate directly?
        - Can you tell me about their role in the budget conferences?

- Can you tell me more about the groups that are represented in resource allocation processes for health systems decision making?
    - [Probes] (age groups, genders, income levels, etc.)

- How are the interests of vulnerable groups represented in the district for resource allocation decisions?
    - [Probes]:
        - If I'm a person living in the village how does the district gain my input when making health-related resource allocation decisions?
        - Who represents these interests?
        - What do you believe are some of the barriers to the participation of vulnerable women in decision making at the village level? At the district?

Now that we've talked about women who are vulnerable, we will concentrate on this group.

Participation: Vulnerable women

- Who are considered vulnerable women in the context of Tororo District from the perspective of the district?

- How are their interests represented at the district level? By whom, are their interests represented at the district level?

- How are vulnerable women or those who represent them identified for the purposes of engaging them in priority setting for health care?

    - What are the strengths of this approach? What are the weaknesses?

- How do these vulnerable women participate in resources allocation decisions at the local level?

Participation: How?

- How should these women be involved in making decisions about the distribution of resources?
    - What are some of the challenges with engaging vulnerable women in resource allocation decisions?

- How can the district facilitate the participation of these women considering [the barriers identified by the participants above]?

- Is there anything further that you wish to add?

Thank you for your participation. Please feel free to contact me if necessary.
