# Supplementary material for: Barriers to Equitable Public Participation in Health-System Priority Setting Within the Context of Decentralization: The Case of Vulnerable Women in a Ugandan District
Source: Int J Health Policy Manag. 2020 Dec 26;11(7):1047–57. doi: 10.34172/ijhpm.2020.256 (PMC9808191; doi:10.34172/ijhpm.2020.256)
Supplement: Supplementary file 3 — Programme For Tororo District Local Government. [file ijhpm-11-1047-s003.pdf]

Supplementary file 3. Programme For Tororo District Local Government

Budget Conference at the Council Chambers on October 24, 2018

TIME	PROGRAMME	PRESENTER	SESSION/CHAIRPERSON
8:30-9:00am	Registration	Budget Desk	Budget Desk
9:00-9:15 am	Opening remarks	Ag District Chairperson	Chairperson for Finance, Planning Administration and Investment Committee
9:15-9:30am	Overview of the Budget conference	CAO	
9:30-9:45am	Administration presentation	D/CAO	
9:45-9:55am	Finance presentation	CFO	
9:55-10:05am	Planning presentation	D/Planner	
10:05-10:15am	Statutory bodies presentation	CTC	
10:15-10:25 am	Internal Audit presentation	DIA	
10:25-11:00 am	Discussion of Finance Planning, Statutory bodies and Internal Audit	All	
11:00-11:15 am	Education presentation	DEO	Chairperson for Health and Education Committee
11:15-11:30 am	Health presentation	DHO	
11:30am-12:00pm	Discussion of Health and Education	All	
12:00-12:45pm	Presentation by development partners 5 minutes each	Representatives of development partners	
12:45pm – 1:15pm	Discussion on the partners presentation	All	
**1:15-2:00pm**	**Lunch**	**Budget Desk**	**Budget Desk**
2:00 – 2:15pm	Production and Marketing	DPO	Secretary for Production and Marketing
2:15 – 2:30pm	Natural Resources	DNRO	
2:30 – 3:00pm	Discussion of Production and natural resources	All	
3:00 - 3:15 pm	Works and technical services	D/Engineer	Chairperson for Works and technical services Committee
3:15 – 3:45pm	Discussion of works	D/Engineer	
3:45 – 4:00pm	Community Based Services	DCDO	Chairperson for Community Based Service's Committee
4:00 - 4:30pm	Discussions of Community Development	All	
4:30- 4:45pm	Way Forward	CAO	
4:45 – 5:00pm	Closure	RDC	
