# Supplementary material for: Barriers to Equitable Public Participation in Health-System Priority Setting Within the Context of Decentralization: The Case of Vulnerable Women in a Ugandan District
Source: Int J Health Policy Manag. 2020 Dec 26;11(7):1047–57. doi: 10.34172/ijhpm.2020.256 (PMC9808191; doi:10.34172/ijhpm.2020.256)
Supplement: Supplementary file 4 — Recommendations to Address Barriers and Enhance Participation for Vulnerable Women in Healthcare Prioritization and Planning Processes. [file ijhpm-11-1047-s004.pdf]

Supplementary file 4. Recommendations to Address Barriers and Enhance Participation for Vulnerable Women in Healthcare Prioritization and Planning Processes

**Category of barrier**	**Barriers to participation**	**Recommendations from the Field**	**Illustrative example**
Financial	Transport (distance/cost)	Hold meetings at the health centers within the community	"If the health center is near it will reduce our distance and costs of traveling." (Japadhola, 49)<br><br>"Use the opportunity of these facilities, they come every other day, we use this opportunity, five to ten minutes and listen to them." (DHMT member)
	Lack of incentives	Provide incentives including transport, allowance, food	"they should make movement for people easy, then they mobilize people for the meetings when they have provided that transport… even if it were UGX1000/- only so that people can use for transport people will accept." (Iteso, 59)
Biomedical and/or health	Illness/Disability	Provide transportation	"[*send*] a boda boda to pick me" (Japadhola, 60)
	Menstruation	Provide adolescent women with female hygiene products	"Absence of pads… when I have pads [*I go*]… if I am sure it is going to come I pad myself and go and attend." (Japadhola, 16)
Knowledge-based	Lack of knowledge (education/literacy)	Hold meetings in local language and/or provide interpreter services	"There are questions they ask in English and if you are not educated and you cannot communicate." (Japadhola, 49)<br><br>"But if it is being done in the native language you will find everybody is interested. But if you speak English... People switch off." (DHMT member)

	Lack of information about participation (rights/opportunities)	Identify an enthusiastic, capable woman from the community sensitize and educated about participation. She would return to collaborate with the community.	"There are right now who are educated. Such educated women should be taken to the district then they will also come back and spread the information to others. So as to empower them." (Iteso, 57)
Motivational	Competing needs/time commitments	Host meetings at times when target populations can attend	"If you organise a meeting in the morning hour…at the time when the women are expected to be in the garden, you will not achieve what you have planned." (Sub-county leader)
	Perceived laziness/disinterest	The women already gather informally, add a formal representative to meetings	"They should bring… meetings to the villages because there are those women who don't attend but when they hear that the district level person who has, come to meet the people, they will come…" (Iteso, 60)
	Lack of feedback/follow through	Strengthen community dialogues/barazas to enhance two-way communication between rural women and local governments	"Personally, my idea is that whatever has been discussed should not remain here it should be put in practice." (Japadhola, 18)<br><br>"What do they have to say about our services? We are the ones always giving them, it is the one way to get feedback from them and I look at it also as a good platform to listen from these vulnerable people, not a mother to just come and listen from you and walk away, they leave when they have something burning, so if we give them that platform, it will also be good." (DHMT member)
Socio-cultural	Lack of decision-making power	Develop and support females within the local governance structures	"Let's empower the women who are in decision-making within the local governance structure…they can take lead in seeing to it that they can define a vulnerable woman. They spearhead the whole process as per creating a difference in the livelihood of this woman sometimes when a woman is speaking to a fellow woman, she will be able to speak out

			compared when she is speaking to a man. He may not ably understand how she feels and what it takes for her to get out of a particular situation" (Sub-county leader)
Structural	Hunger	Organizers provide some type of snack or lunch	"Right now, the hunger is smoking hot, they should give food." (Iteso, 70)
	Poverty	Social assistance and development programs to target poverty and daily living expenses ie, school fees, adequate housing, skills training	"I think that the only way that can be done is motivation if they can be motivated or maybe put a learning center at a certain place like in parishes where they can go, they say if you don't know how to write, we don't know how to do what, we need all of to come here. When they go there maybe somebody to train them on crafts to train them on hat maybe the tailoring machine so that can help them to do what, can help integrate them, then they also begin participating" (Sub-county leader)
